# A machine learning-based score for precise echocardiographic assessment of cardiac remodelling in hypertensive young adults

**DOI:** 10.1093/ehjimp/qyad029

**Published:** 2023-09-27

**Authors:** Maryam Alsharqi, Winok Lapidaire, Yasser Iturria-Medina, Zhaohan Xiong, Wilby Williamson, Afifah Mohamed, Cheryl M J Tan, Jamie Kitt, Holger Burchert, Andrew Fletcher, Polly Whitworth, Adam J Lewandowski, Paul Leeson

**Affiliations:** Oxford Cardiovascular Clinical Research Facility, Division of Cardiovascular Medicine, Radcliffe Department of Medicine, University of Oxford, John Radcliffe Hospital, Oxford OX39DU, UK; Department of Cardiac Technology, College of Applied Medical Sciences, Imam Abdulrahman Bin Faisal University, Dammam, Saudi Arabia; Oxford Cardiovascular Clinical Research Facility, Division of Cardiovascular Medicine, Radcliffe Department of Medicine, University of Oxford, John Radcliffe Hospital, Oxford OX39DU, UK; Neurology and Neurosurgery Department, Montreal Neurological Institute, Montreal, Canada; Oxford Cardiovascular Clinical Research Facility, Division of Cardiovascular Medicine, Radcliffe Department of Medicine, University of Oxford, John Radcliffe Hospital, Oxford OX39DU, UK; Oxford Cardiovascular Clinical Research Facility, Division of Cardiovascular Medicine, Radcliffe Department of Medicine, University of Oxford, John Radcliffe Hospital, Oxford OX39DU, UK; Oxford Cardiovascular Clinical Research Facility, Division of Cardiovascular Medicine, Radcliffe Department of Medicine, University of Oxford, John Radcliffe Hospital, Oxford OX39DU, UK; Department of Diagnostic Imaging and Applied Health Sciences, Faculty of Health Sciences, Universiti Kebangsaan Malaysia, Kuala Lumpur, Malaysia; Oxford Cardiovascular Clinical Research Facility, Division of Cardiovascular Medicine, Radcliffe Department of Medicine, University of Oxford, John Radcliffe Hospital, Oxford OX39DU, UK; Oxford Cardiovascular Clinical Research Facility, Division of Cardiovascular Medicine, Radcliffe Department of Medicine, University of Oxford, John Radcliffe Hospital, Oxford OX39DU, UK; Oxford Cardiovascular Clinical Research Facility, Division of Cardiovascular Medicine, Radcliffe Department of Medicine, University of Oxford, John Radcliffe Hospital, Oxford OX39DU, UK; Oxford Cardiovascular Clinical Research Facility, Division of Cardiovascular Medicine, Radcliffe Department of Medicine, University of Oxford, John Radcliffe Hospital, Oxford OX39DU, UK; Oxford Cardiovascular Clinical Research Facility, Division of Cardiovascular Medicine, Radcliffe Department of Medicine, University of Oxford, John Radcliffe Hospital, Oxford OX39DU, UK; Oxford Cardiovascular Clinical Research Facility, Division of Cardiovascular Medicine, Radcliffe Department of Medicine, University of Oxford, John Radcliffe Hospital, Oxford OX39DU, UK; Oxford Cardiovascular Clinical Research Facility, Division of Cardiovascular Medicine, Radcliffe Department of Medicine, University of Oxford, John Radcliffe Hospital, Oxford OX39DU, UK

**Keywords:** machine learning, semi-supervised, cardiac remodelling score, echocardiography, hypertension, young adults

## Abstract

**Aims:**

Accurate staging of hypertension-related cardiac changes, before the development of significant left ventricular hypertrophy, could help guide early prevention advice. We evaluated whether a novel semi-supervised machine learning approach could generate a clinically meaningful summary score of cardiac remodelling in hypertension.

**Methods and results:**

A contrastive trajectories inference approach was applied to data collected from three UK studies of young adults. Low-dimensional variance was identified in 66 echocardiography variables from participants with hypertension (systolic ≥160 mmHg) relative to a normotensive group (systolic < 120 mmHg) using a contrasted principal component analysis. A minimum spanning tree was constructed to derive a normalized score for each individual reflecting extent of cardiac remodelling between zero (health) and one (disease). Model stability and clinical interpretability were evaluated as well as modifiability in response to a 16-week exercise intervention. A total of 411 young adults (29 ± 6 years) were included in the analysis, and, after contrastive dimensionality reduction, 21 variables characterized >80% of data variance. Repeated scores for an individual in cross-validation were stable (root mean squared deviation = 0.1 ± 0.002) with good differentiation of normotensive and hypertensive individuals (area under the receiver operating characteristics 0.98). The derived score followed expected hypertension-related patterns in individual cardiac parameters at baseline and reduced after exercise, proportional to intervention compliance (*P* = 0.04) and improvement in ventilatory threshold (*P* = 0.01).

**Conclusion:**

A quantitative score that summarizes hypertension-related cardiac remodelling in young adults can be generated from a computational model. This score might allow more personalized early prevention advice, but further evaluation of clinical applicability is required.

## Graphical Abstract

**Graphical abstract:**
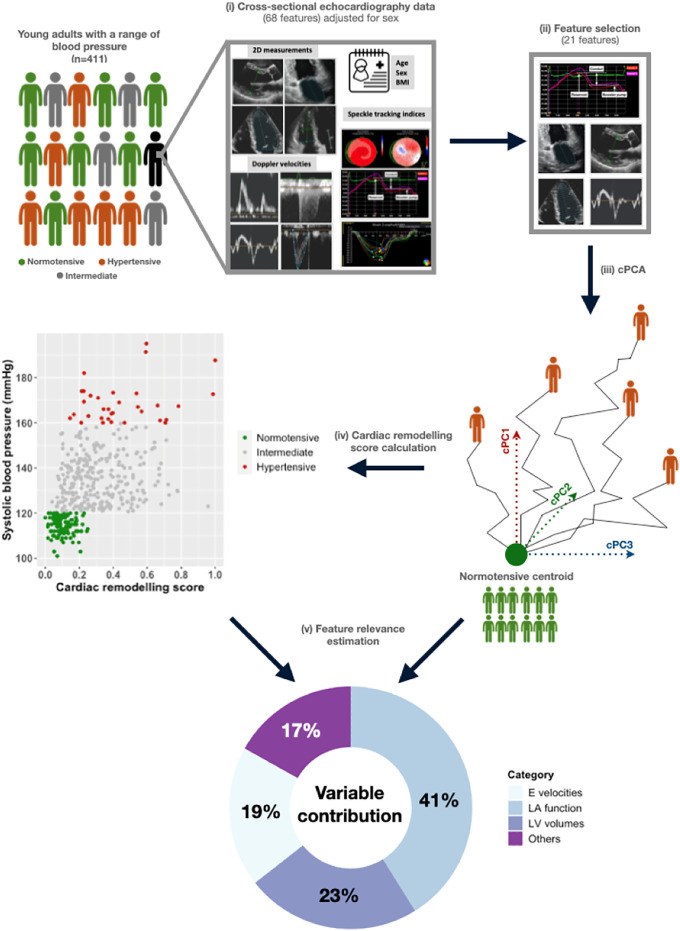

## Introduction

Hypertension in young adulthood is associated with an increased risk of early stroke and cardiovascular disease.^1–3^ However, the natural history of hypertension in young adults is unpredictable, and there is caution about starting interventions as they may need to be continued for many decades.^4,5^ Evidence of left ventricular hypertrophy is a trigger for pharmacological treatment, as end-organ changes identify individuals most physiologically vulnerable to higher blood pressures.^6–9^ Identification of individuals with earlier signs of cardiac remodelling may therefore also be of value to identify those who should be targeted for preventive interventions. However, early cardiac remodelling is characterized by changes in multiple cardiac parameters, including emerging indices such as left atrial strain, making it difficult to identify a simple summary marker that could be evaluated in studies.^10–12^

A novel machine learning approach is now available that can integrate high-dimensional cross-sectional data to identify pseudo-temporal patterns and generate a summative score of disease progression. This has previously been applied to gene expression data to order cancer disease severity^13–15^ and, subsequently, within a contrastive trajectory inference (cTI) algorithm,^16^ to identify enriched neuroimaging patterns in Alzheimer’s disease.^16,17^ We have applied this approach to cardiac imaging data from young adults to evaluate whether it can characterize patterns of cardiac remodelling related to hypertension. We tested the internal stability and validity of the resulting derived cardiac remodelling score, investigated the clinical validity of the score by studying how it related to longitudinal patterns of known hypertensive cardiac remodelling, and studied how the score changed over a 16-week exercise intervention.

## Methods

### Study data set

#### Study population

The study data set comprised of cross-sectional data collected up to March 2020 in young adults with a range of blood pressures from three ethically approved studies: (i) the Young Adult Cardiovascular Health sTudy (YACHT), (ii) Trial of Exercise to Prevent HypeRtension in young Adults (TEPHRA), and (iii) Hypertension management in Young adults Personalised by Echocardiography and clinical Outcomes (HyperEcho). A written informed consent was obtained from all eligible participants prior to their participation. The eligibility criteria for each study are available in the Supplementary Materials.

The YACHT study (NCT02103231) was a single-centre, observational case-control study, started in August 2014 and completed in May 2016.^18^ The study was approved by the South Central Berkshire Research Ethics Committee (Reference 14/SC/0275).

The TEPHRA study (NCT02723552) was a single-centre, two-arm, and parallel randomized controlled (1:1) trial, started in June 2016 and completed in January 2020.^18,19^ All participants underwent a baseline study visit for detailed assessment of cardiovascular structure and function. Eligible participants were randomized to either a 16-week exercise intervention arm or control arm. Participants who were randomised to the exercise intervention arm were provided with a gym membership to complete three supervised aerobic exercise sessions (60 min each) per week and for 16 weeks. The control arm participants were advised to maintain their usual physical activity levels. After 16 weeks of randomization, all participants attended their second visit for a follow-up cardiovascular assessment.^18^ Trial of Exercise to Prevent HypeRtension in young Adults was approved by the Oxford B Research Ethics Committee (Reference 16/SC/0016).

The HyperEcho study (NCT03762499) is a multi-centre, longitudinal, observational study of hypertensive patients aged between 18 and 40 years old and referred to a hypertension clinic in England to manage their blood pressure. The study started in October 2018 and was approved by the South West—Frenchay Research Ethics Committee (Reference 18/SW/0188). This study is still ongoing, and participants recruited before March 2020 were included in this analysis.

Participants included in this work were recruited from five sites within England, (i) Oxford University Hospitals, (ii) George Eliot Hospital, (iii) High Wycombe Hospital, (iv) Broomfield Hospital, and (v) Nottingham Hospital. The three data sets were combined after independent data processing and cleaning. The results of TEPHRA and YACHT studies have demonstrated there are specific patterns of cardiac remodelling related to prematurity independent of blood pressure.^20–22^ Therefore, participants with known history of premature birth were excluded to ensure there was not disproportionately represented in the cohort used to build the model. Participants with missing data of >30% were also excluded.

#### Clinical data

Demographic data including age, sex, height, weight, and body mass index were collected from all enrolled participants at their baseline visit. Resting blood pressure measurements were obtained using a digital blood pressure monitor (GE Dinamap V100, GE Healthcare, Chalfont St. Giles, UK) to record three consecutive blood pressure readings on the left arm with a minute apart. The last two measurements were averaged and included in the analysis.

#### Echocardiography

The echocardiography assessment was performed in the Oxford Cardiovascular Clinical Research Facility Echocardiography Core Lab. A comprehensive transthoracic 2D echocardiography scan was performed for all participants at the baseline visit using Philips EPIC 7C or Philips iE33 echocardiography ultrasound machines (Philips Healthcare, Surrey, United Kingdom) and the xMATRIX array transducer (X5-1). All images were acquired according to the British Society of Echocardiography guidelines in image acquisition and optimization.^23^ Image acquisition and interpretation were performed following the same standards and latest echocardiography guidelines in the three clinical studies. Conventional image analysis was completed offline following the latest guidelines for structural and functional cardiac assessment,^24^ using Philips IntelliSpace Cardiovascular (ISCV) 2.1 (Philips Healthcare Informatics, Belfast, Ireland), and TomTec Image Arena 4.6 software (Chicago, IL, USA) was used to perform 2D left ventricular and left atrial speckle tracking analysis following the European Association of Cardiovascular Imaging (EACVI) recommendations.^25^ Additional echocardiography scan was performed for TEPHRA participants in their follow-up visit after the 16-week exercise intervention.

#### Sixteen-week exercise intervention

Trial of Exercise to Prevent HypeRtension in young Adults participants had taken part in a 16-week exercise intervention comprising three aerobic training sessions per week, aiming for 60 min of exercise at 60–80% peak heart rate measured at baseline. Participants were encouraged to attend supervised sessions at the gym (Brookes Sport) or supported in a remote exercise intervention programme. Compliance was assessed from wrist-worn heart rate and activity monitors (Fitbit Charge HR) as well as records of exercise sessions attended and activity from the wrist-worn activity monitor. Participants who completed 80% of planned exercise sessions were considered compliant.^19^

### Model development and internal testing

Model development was performed in the MATLAB R2019b programming environment (Mathworks Inc., Natick, MA, USA) using the cTI algorithm (https://www.neuropm-lab.com/neuropm-box.html).^16,17^ The combined data set of clinical and echocardiography data obtained at the baseline visit from the three clinical studies (YACHT, TEPHRA, and HyperEcho) was used for the model development. Prior to model development, participants and variables with more than 30% missing data were excluded from the analysis, and the remaining missing data were imputed using trimmed scores regression (TSR). Participants were classified based on resting blood pressure measures as normotensive group (participants with systolic blood pressure < 120 mmHg and not on anti-hypertension medication) and hypertensive group (participants with systolic blood pressure ≥ 160 mmHg). All remaining participants were allocated in the intermediate group which did not contribute to the contrastive dimensionality reduction. As the outcomes of the cTI method is strongly dependent on the definition of the hypertensive and normotensive populations,^17^ these definitions must consider the biological process of hypertension ensuring that the hypertensive participants have pathological patterns related to hypertension, while the normotensive group consists of pathology-free participants only. Following this classification, the cTI algorithm^16,17^ was then applied to the echocardiography data using the five-step process, illustrated in Supplementary data online, *Figures S1 and S2*. Briefly, the stages comprised the following: (i) data adjustment for sex using additive linear models with pair-wise interactions^26^; (ii) unsupervised feature selection based on comparison of participant and neighbourhood variance^27^; (iii) data visualization and exploration using a contrasted principal component analysis (cPCA) tool to identify enriched, non-linear, low-dimensional patterns in the participants defined as ‘hypertensive’ relative to participants classed as ‘normotensive’.^28^ The trade-off between the hypertensive and normotensive variances was represented by a contrast parameter (α), which was automatically selected by the algorithm based on the subspace that maximizes the clustering tendency in the hypertensive data; (iv) construction of a minimum spanning tree (MST)^17^ using the distances between each sample in the reduced dimensional space to calculate the pseudo-temporal cardiac remodelling scores as the shortest distance value along the MST from any participant to the ‘normotensive’ centroid and normalize the value to be between zero (healthy state) to one (disease state); and (v) estimation of feature relevance to quantify total contribution to the obtained reduced representation space.^29^

Model stability and internal validity were assessed within a five-fold cross-validation test with 20% hypertensive and normotensive participants hold out in each fold. Internal model validity was assessed on the ability of the derived score to differentiate between ‘normotensive’ and ‘hypertensive’ participants with a *P*-value of ≤0.05 in an independent samples *t*-test. A receiver operating characteristic (ROC) analysis was performed to assess the model sensitivity and specificity at different thresholds. Stability was determined from the root mean squared deviation (RMSD) of differences between repeated and original values for individuals between each fold, with a value of <0.2 considered acceptable. After developing this model, we have compared the model performance with another model, in which left atrial strain indices were excluded. A description of the model development of the later model is available in the Supplementary Methods.

### Clinical validation

Statistical Software R 4.0.2 was used for the clinical validation assessment via two approaches. The first approach was to assess changes of individual variables throughout the disease progression by testing the changes of the identified highly contributed variables. The pattern of changes in key echocardiographic variables across the range of scores was visualized by plotting re-scaled values of variables against the score in line graphs and a heat map. To create the heat map, subjects with a score of ≤0.25 were defined as Group 1, and then each subsequent consecutive group of 20 subjects were ranked from 2 up to 10. The second approach was to test the effect of 16-week exercise intervention, which involves a sub-group of the cohort (TEPHRA participants only) using the data from the second visit of TEPHRA participants who were randomized for the exercise intervention arm. Pearson correlation and linear regression tests were used to test linear associations between the change in scores after a 16-week exercise intervention and fitness variables including ventilatory aerobic threshold and the number of active days in the gym. Comparisons between the compliant and non-compliant participants to the exercise intervention were performed using two-sided, independent samples Student’s *t*-tests. A *P*-value of ≤0.05 and a 95% confidence interval were used to indicate statistical significance.

## Results

### Baseline clinical characteristics

Between August 2014 and March 2020, 542 young adults were enrolled into the three studies, of which 131 participants were excluded from this analysis (*n* = 117 participants with history of premature birth and *n* = 14 participants with >30% missing data). A flow diagram of the study population is available in the Supplementary data online, *Figure S3*. About 3% of the overall data were imputed prior to the model development. Demographic description of the 411 participants is presented in *Table 1*. Mean age was 28.9 ± 5.7 years, and 51.6% were male with an average body mass index of 26.3 ± 5. Blood pressure ranged within the group from 101 to 195 mmHg systolic blood pressure and from 56 to 125 mmHg diastolic blood pressure. The clinical characteristics for each group are demonstrated in Supplementary data online, *Table S1*.

**Table 1:** Baseline clinical characteristics for the study cohort

	Study cohort *n* = 411
Age	28.9 ± 5.7 (22)
Male, *n* (%)	209 (51.6)
Height (cm)	173 ± 10.03 (57)
Weight (kg)	79.2 ± 18.5 (135.4)
Body mass index (kg/m^2^)	26.3 ± 5.01 (32.3)
Body surface area (m^2^)	1.9 ± 0.2 (1.1)
Systolic blood pressure (mmHg)	132.2 ± 16.6 (94)
Diastolic blood pressure (mmHg)	81.7 ± 12.8 (68.7)
Cholesterol level (mmol/L)	4.5 ± 1.1 (9.4)
HDL level (mmol/L)	1.3 ± 0.3 (2.6)
LDL level (mmol/L)	2.7 ± 0.8 (5)
Triglycerides level (mmol/L)	1.2 ± 0.9 (5.03)
Cholesterol to HDL ratio	3.5 ± 1.2 (10.7)
Smokers, *n* (%)	45 (11.59)
On anti-hypertension medication, *n* (%)	124 (31.47)

Numeric data are presented as mean ± standard deviation and (range), and categorical data are presented as number of participants and (percentage).

### Model development and variable contributions

**Figure 1:**
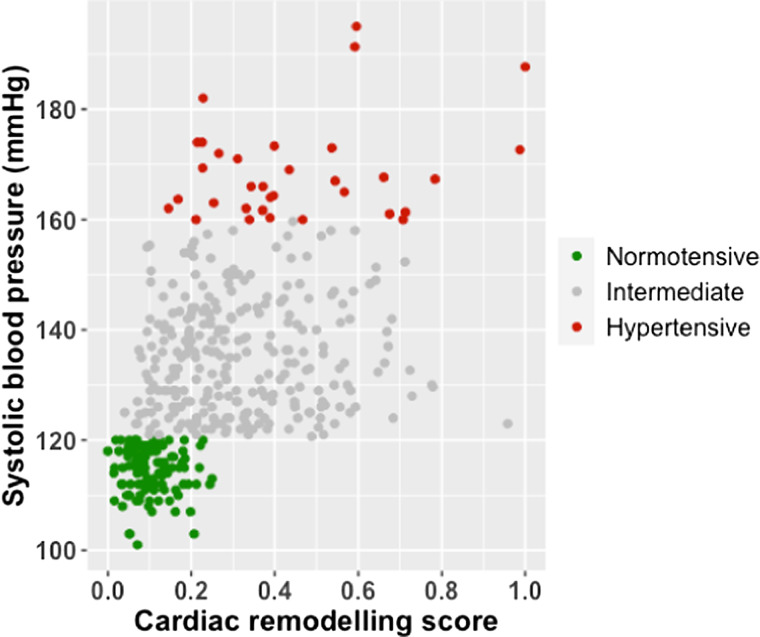
A scatter plot to demonstrate the relationship between the derived cardiac remodelling scores and resting systolic blood pressure for all participants.

We included 66 echocardiography variables along with age and body mass index in the model. To account for the fact that echocardiography parameters vary by body size, body mass index was included as an independent variable in the model development. The included variables are listed in *Table 2* and represent echocardiography metrics that comprehensively describe the cardiac structure and function using 2D measures, Doppler velocities, and speckle tracking indices. A summary of echocardiography characteristics for the participants is provided in *Table 3*, and the echocardiography characteristics for each group are demonstrated in Supplementary data online, *Table S2*. The relationship between the cardiac remodelling score and resting systolic blood pressure for all participants is shown in *Figure 1*. After the contrastive dimensionality reduction, 21 variables that contributed >80% of the variance within the developed model were identified. These variables were grouped into three categories; (i) measures of left atrial structure and function, (ii) left ventricular volumes, and (iii) Doppler velocities. Supplementary data online, *Figure S4* illustrates the contribution of each of these three categories to the model compared to the remaining 47 variables. The algorithmically selected α value for the model was 22.57.

**Table 2:** Variables included for the computational disease progression model development

List of variables	
1. Age (years)	35. Lateral *a*′ velocity (cm/s)
2. Body mass index (kg/m^2^)	36. Septal *s*′ velocity (cm/s)
3. Heart rate (bpm)	37. Septal *e*′ velocity (cm/s)
4. Interventricular septum (cm)	38. Septal *a*′ velocity (cm/s)
5. LV internal diastolic dimension (cm)	39. *e*′_average_ (cm/s)
6. LV posterior wall thickness (cm)	40. *E*/*e*′_lateral_
7. LV internal systolic dimension (cm)	41. *E*/*e*′_septal_
8. LV ejection fraction, Teichholz (%)	42. *E*/*e*′_average_
9. LV outflow tract (cm)	43. Aortic valve max velocity (cm/s)
10. LV relative wall thickness	44. LVOT velocity time integral (cm)
11. LV mass (g)	45. Pulmonary valve max velocity (cm/s)
12. LV mass index (g/m^2^)	46. Pulmonary artery acceleration time (s)
13. LV 4-ch end diastolic volume (ml)	47. RV basal dimension (cm)
14. LV 4-ch end systolic volume (ml)	48. RV mid dimension (cm)
15. LV 4-ch ejection fraction (%)	49. RV length (cm)
16. LV 4-ch stroke volume (ml)	50. RA volume (ml)
17. LV 2-ch end diastolic volume (ml)	51. Tricuspid regurgitation max velocity (cm/s)
18. LV 2-ch end systolic volume (ml)	52. TAPSE (cm)
19. LV 2-ch ejection fraction (%)	53. RV *s*′ velocity (cm/s)
20. LV 2-ch stroke volume (ml)	54. RV *e*′ velocity (cm/s)
21. LV biplane end diastolic volume (ml)	55. RV *a*′ velocity (cm/s)
22. LV biplane end systolic volume (ml)	56. Isovolumetric contraction time (s)
23. LV biplane ejection fraction (%)	57. Isovolumetric relaxation time (s)
24. LV biplane stroke volume (ml)	58. Ejection time (s)
25. LV biplane cardiac output (ml/min)	59. LV global longitudinal strain (%)
26. LA 4-ch volume (ml)	60. LA peak longitudinal strain, 4-ch reservoir (%)
27. LA 2-ch volume (ml)	61. LA peak contraction strain, 4-ch booster pump (%)
28. LA biplane volume (ml)	62. LA 4-ch conduit (%)
29. Mitral valve *E* velocity (cm/s)	63. LA peak longitudinal strain, 2-ch reservoir (%)
30. Mitral valve *A* velocity (cm/s)	64. LA peak contraction strain, 2-ch booster pump (%)
31. *E*/*A*	65. LA 2-ch conduit (%)
32. Mitral valve deceleration time (s)	66. LA peak longitudinal strain—biplane reservoir (%)
33. Lateral *s*′ velocity (cm/s)	67. LA peak contraction strain—biplane booster pump (%)
34. Lateral *e*′ velocity (cm/s)	68. LA biplane conduit (%)

LV, left ventricle; LA, left atrium; 4-ch, four-chamber; 2-ch, two-chamber; LVOT, left ventricular outflow tract; RV, right ventricle; RA, right atrium; TAPSE, tricuspid annular plane systolic excursion.

**Table 3:** Echocardiography characteristics for the study cohort

	Study cohort *n* = 411
Heart rate (bpm)	64.61 ± 11.53
*Left ventricular structure and function*	
Diastolic diameter (cm)	4.71 ± 0.48
Systolic diameter (cm)	3.10 ± 0.44
Interventricular septum thickness (cm)	0.89 ± 0.20
Inferolateral (posterior) wall thickness (cm)	0.93 ± 0.18
Relative wall thickness	0.40 ± 0.09
Mass index (g/m^2^)	72.38 ± 18.17
Biplane end diastolic volume (ml)	99.48 ± 25.76
Biplane end systolic volume (ml)	37.00 ± 11.79
Biplane ejection fraction (%)	63.09 ± 5.57
Biplane stroke volume (ml)	62.44 ± 16.28
Mitral valve *E* velocity (cm/s)	78.81 ± 15.82
Mitral valve *A* velocity (cm/s)	53.38 ± 12.59
*E*/*A* ratio	1.55 ± 0.44
Mitral valve deceleration time (s)	0.19 ± 0.04
Lateral *e*′ velocity (cm/s)	15.43 ± 3.92
Septal *e*′ velocity (cm/s)	10.67 ± 2.41
*E*/*e*′_Lateral_ (cm/s)	5.41 ± 1.70
*E*/*e*′_Septal_ (cm/s)	7.67 ± 1.99
Global longitudinal strain (%)	−20.34 ± 2.28
*Left atrial structure and function*	
Biplane left atrial volume (ml)	40.55 ± 11.99
Reservoir function—peak longitudinal strain (%)	36.75 ± 7.67
Booster pump function—peak contraction strain (%)	9.71 ± 5.26
Conduit function—the difference (%)	26.95 ± 7.63
*Right heart structure and function*	
RV basal diameter (cm)	3.54 ± 0.52
RV mid diameter (cm)	2.57 ± 0.54
RV length (cm)	7.00 ± 0.88
TAPSE (cm)	2.16 ± 0.34
RV s′ velocity (cm/s)	12.57 ± 1.94
RA volume (ml)	37.20 ± 12.68

Data are presented as mean ± standard deviation.

Bpm, beats per minute; RV, right ventricle; RA, right atrium; TAPSE, tricuspid annular plane systolic excursion.

The results of the internal validation demonstrated the model reached acceptable criteria for validity by separating hypertensive from normotensive participants sufficiently. The mean cardiac remodelling score for the normotensive group was lower compared to the hypertensive group (0.2 ± 0.17 vs. 0.4 ± 0.21, *P* < 0.0001). Using an optimal threshold of 0.21 for the score derived from ROC analysis, our model differentiates hypertensives from normotensives with a sensitivity of 94% and specificity of 94.6% (97.5% AUC). The results of the five-fold cross-validation demonstrated the model maintained acceptable stability with the RMSD for differences between repeated scores for the same individuals being 0.1 ± 0.002. The model developed without left atrial indices showed a lower precision for class separation of hypertensives from normotensives with a sensitivity of 89% and specificity of 88%, based on an optimized threshold of 0.31 for this new model, compared to the original model.

### Clinical validation assessment

The results of the clinical validation assessment of the derived cardiac remodelling scores were demonstrated in two sections.

#### Changes of individual variables throughout the disease progression

**Figure 2:**
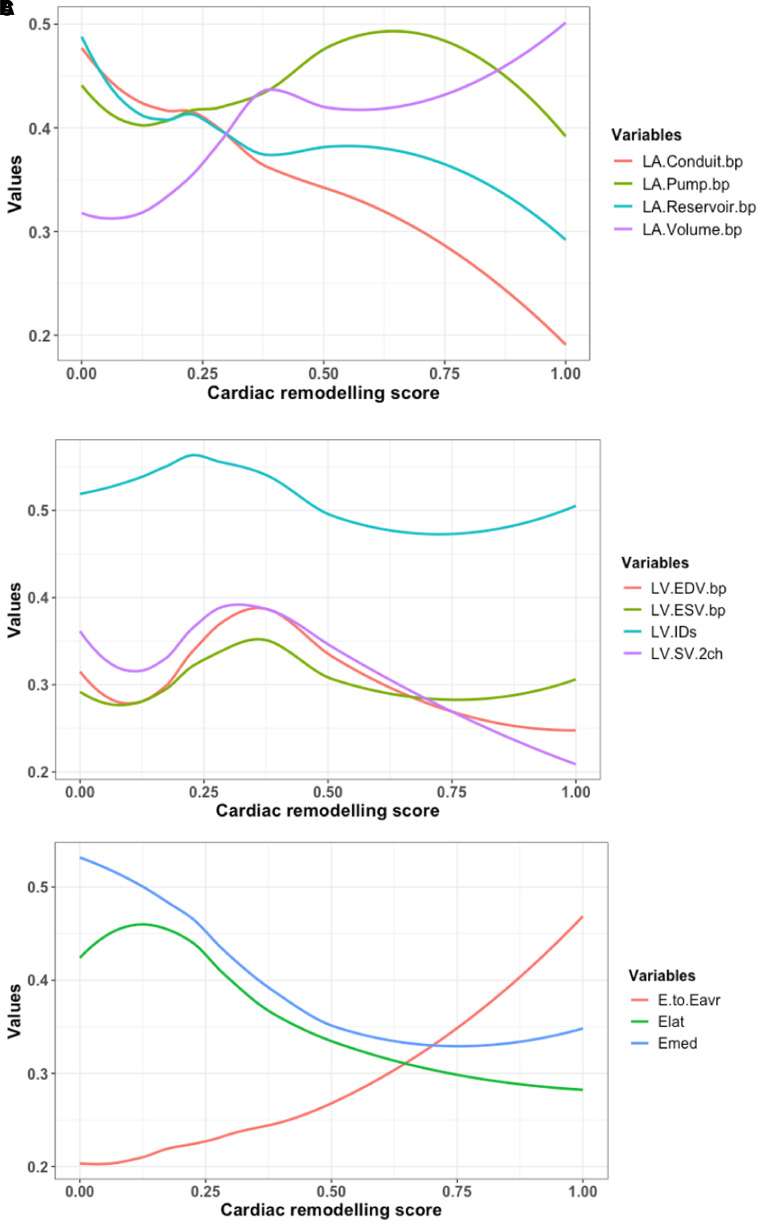
The pattern of remodelling in individual variables and the continuous relationship between the cardiac remodelling score and left atrial structure and function (*A*), left ventricular measures (*B*), and Doppler velocities (*C*). All values were re-scaled from zero to one to allow between-variable comparisons.

**Figure 3:**
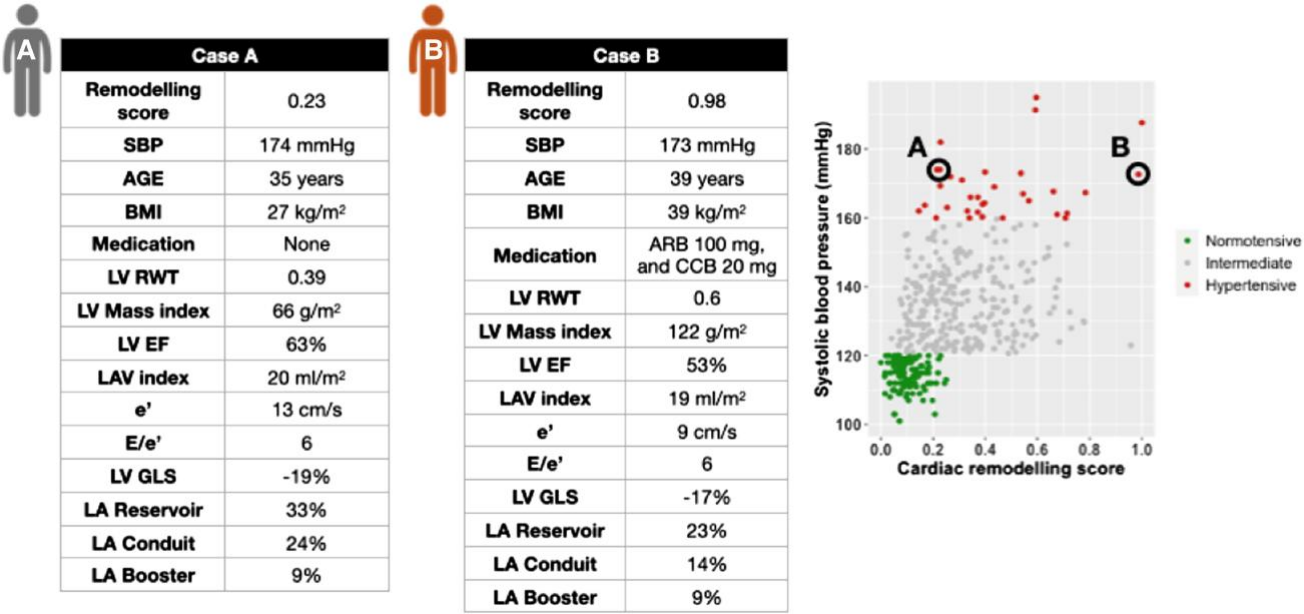
Individual clinical and echocardiographic characteristics for two cases with similar systolic blood pressure but different cardiac remodelling score. Case A illustrates a hypertensive participant (SBP 174 mmHg) with low cardiac remodelling score (0.23), while Case B shows the characteristics for another hypertensive participant with more advanced stage of cardiac remodelling (score is 0.98).

The continuous relationship between the derived score and echocardiographic variables was studied. Left atrial structure and function, left ventricular measures, and Doppler velocities are illustrated in *Figure 2A–C*, respectively. Left atrial conduit and reservoir function reduce as the cardiac remodelling score increases but with a steeper reduction in the conduit function (*Figure 2A*), and, interestingly, the booster pump function appears to have a biphasic pattern of remodelling. While the left atrial volume increases rapidly until the score reaches 0.4 and then increases at a slower rate with a maximum increase at score one. Panel B demonstrates the changes in left ventricular systolic diameter and left ventricular volumes. All measures have the same pattern of changes through the spectrum of the cardiac remodelling score with their peak at 0.4 except the systolic diameter, which peaks earlier at 0.25. The change in Doppler velocities is shown in *Figure 2C* with a steep increase of *E*/*e*′ ratio after 0.5 with similar pattern of reduction in the lateral and medial *e*′ velocities. Two cases of similar systolic blood pressure measures, but different cardiac remodelling scores are presented in *Figure 3*. Assessment of the pattern of change displayed as a heat map for each contributing variable is provided in Supplementary data online, *Figure S5*.

#### The effect of 16-week exercise intervention

**Figure 4:**
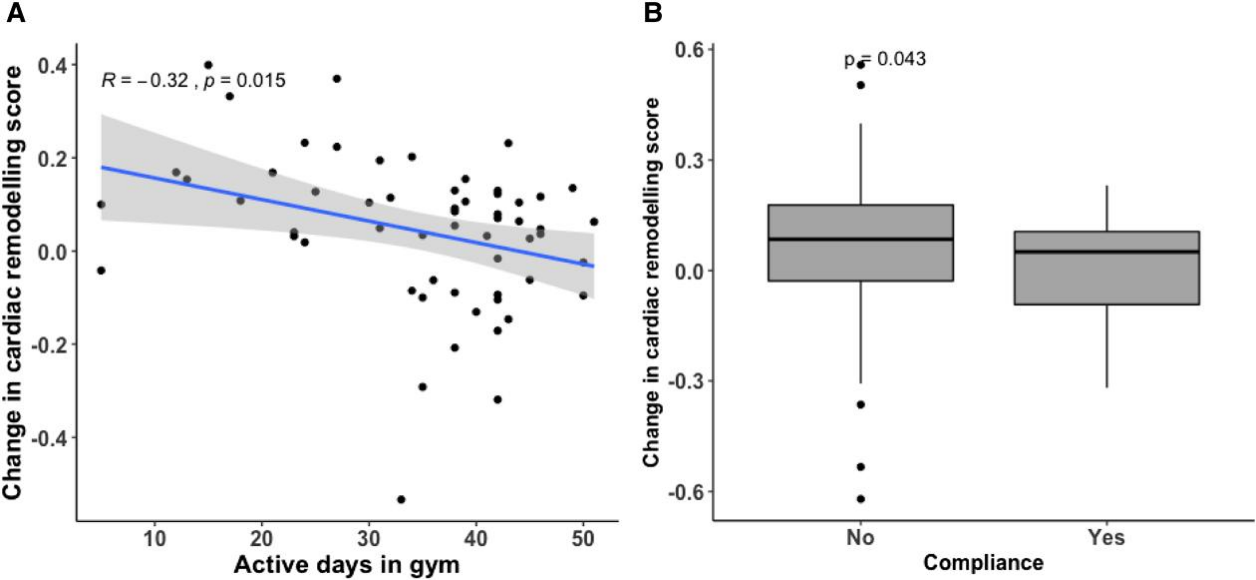
The reduction in the cardiac remodelling score was correlated with a higher number of active days spent at the gym (*P* = 0.015) as shown in *A*. *B* illustrates that compliant participants who attended at least 80% of the exercise intervention had improved their score compared to the non-compliant participants (*P* = 0.043).

The modifiability of the score was assessed based on data from a sub-group (*n* = 60) who underwent a 16-week exercise intervention, mean systolic blood pressure ranged from 110 to 156 mmHg. For these participants, a second cardiac remodelling score was generated from their follow-up echocardiography data. A comparison of echocardiography data between pre- and post-interventions are presented in Supplementary data online, *Table S3*. There were no groups differences in the score and individual parameters of cardiac structure and function post-intervention (*P* = 0.278); however, reduction in the score post-intervention was associated with an increase in the ventilatory threshold levels over the intervention (β=−0.014, *P* = 0.01). Further, the change in the derived cardiac remodelling score post-intervention was correlated with the number of active days participants spent at the gym (*P* = 0.01) as illustrated in *Figure 4A*. *Figure 4B* demonstrates compliant participants, who attended 80% of the exercise intervention sessions, had larger changes in the score than non-compliant participants (*P* = 0.04).

## Discussion

In this study, we developed a computational model of the cardiac alterations of hypertension in young adults and used this to generate a reproducible summary score for an individual to describe their degree of cardiac remodelling. The derived score accurately characterized expected patterns of remodelling and could be modified by an individual when participating in, and fully compliant with, an exercise intervention.

We have demonstrated the first successful application within the cardiovascular disease of a computational method that has previously been applied to neurodegenerative conditions, Huntington disease,^16^ and cancer.^14,15^ Due to the non-linear nature of cardiac remodelling in hypertension, it has been challenging to study the longitudinal changes across multiple different cardiac variables without frequently sampled follow-up data over a long time period.^30^ The cTI tool uses non-linear modelling to generate the pseudo-temporal cardiac remodelling scores and has achieved better performance compared to other dimensionality reduction approaches, such as traditional PCA and novel non-linear Uniform Manifold Approximation and Projection.^16^ Unlike the traditional data exploration methods (i.e. PCA), the cTI approach allows to identify low-dimensional patterns that are enriched in the hypertensive group relative to the normotensive group by controlling the effects of characteristic patterns in the normotensives using the cPCA tool.^28^

Several recent studies have proposed using combinations of echocardiography variables to better describe disease pathology.^31,32^ For example, Katz *et al.*^31^ applied machine learning tools to combine 47 continuous echocardiography, clinical, and laboratory variables to cluster hypertensive patients into distinct groups to assess the benefit from targeted treatment plans. Nevertheless, the application of the novel non-linear cTI method in our model provided additional information that allowed us to study the trajectories of cardiac remodelling from health to advanced stages of hypertension using cross-sectional data. The strength of this study also lies in the combination of echocardiography features, including 2D images, Doppler velocities, and speckle tracking features in this pseudo-temporal cardiac remodelling model. Although this model was based on a single ‘snapshot’ of individuals, the derived score represents the time progression of hypertension relative to the normal point, which is referred to the normotensive centroid.

The outcomes of the cTI method are highly influenced by the definition of the hypertensive and normotensive populations,^17^ hence our selection of normotensive participants with optimal systolic blood pressure (<120 mmHg) without prior history of hypertension or anti-hypertension medication as the background group and our use of a higher threshold of ≥160 mmHg for definition of hypertension.^8,9^ The remaining participants with systolic blood pressure between 120 and 160 mmHg provided variance to the model but did not contribute to the contrastive dimensionality reduction. Reassuringly, when assigned scores, they fitted the expected pattern of cardiac changes for an intermediate group.^17^

To investigate clinical validity of the score, we studied whether the score accurately reflected expected patterns of changes in multiple echocardiography variables related to hypertension. For atrial parameters, the data demonstrated that across the score, left atrial reservoir and conduit function reduce, while booster pump function increases initially and then reduces in more severe disease, consistent with previous studies that report temporary enhancement in booster pump function during early stages of hypertension^33,34^ and prognostic value of left atrial phasic function in hypertension.^35^ Furthermore, left ventricular volumes showed a pattern of reduction as the disease advances which has been widely explained due to the increase in wall thickness according to Laplace’s Law.^30^ Although signs of left ventricular hypertrophy secondary to hypertension has been well studied and linked with poor prognosis later in life,^36^ wall thickness variables and left ventricular mass contributions were not as significant as the functional diastolic variables in our model. The reason could be because of that our cohort consists of young age participants with relatively a short exposure period of hypertension, in which left ventricular hypertrophy has not been developed yet.^33^ Other factors such as presence of coronary disease, diabetes, and exercise training are known to impact cardiac remodelling, and the degree to which these factors vary cardiac remodelling score for an individual beyond the impact of blood pressure will require further investigation.

We have also demonstrated that the early complex cardiac remodelling that would be expected to be induced by an exercise intervention can be tracked through use of the score. Although at a group level the score did not significantly change after the 16-week exercise intervention, this trial also demonstrated that aerobic exercise in isolation does not have an impact on blood pressure level.^19^ However, in those who were fully compliant with the intervention, assessed based on days in the gym, and achieved an objective improvement in their fitness levels, the score reduced. Previous studies demonstrated that the level of adherence and compliance to exercise sessions are associated with more sustained long-term benefits in controlling blood pressure.^37,38^

Our study has limitations. First, although our computational model was adjusted for sex, a larger cohort of young adults may allow individual male and female models to be developed or study of ethnic variation in remodelling patterns. Due to the small sample size, there were relatively fewer participants with high score compared to those with low score, which might introduce a level of bias as the findings are likely to be influenced by these few participants. Second, the majority of participants (>90%) had been recruited at a single centre, which might introduce sources of bias in our findings. Third, some of the variables that were identified as important echocardiography variables relevant to hypertension progression in young adults are not routinely obtained in clinical practice (e.g. left atrial strain indices). This, therefore, could limit the applicability of our results to real-world practice. However, following development of the original model, we assessed the impact of excluding left atrial strain indices on model performance. Although there was a drop in precision based on the internal validation, this was not large and may be clinically acceptable. Future work to identify the optimal echocardiography features for inclusion in a clinically translatable and acceptable model will be worthwhile. Finally, the current sample was insufficient to conduct a completely independent holdout testing data set, and internal validation was performed using a five-fold cross-validation test as well as additional follow-up echocardiography data for a sub-group of the cohort. Further independent validation will be of value in new data sets with the clinically recommended set of echocardiography variables.

In this study, we show that a complex pattern of remodelling described by multiple cardiac parameters generated by echocardiography can be simplified into a single score. This simple score may help to identify individuals with early stages of hypertension-related cardiac remodelling, which may be of value for early prevention of end-organ damage.

## Supplementary Material

qyad029_Supplementary_Data

## Data Availability

De-identified participant data that underlie the results reported in this article are available to researchers on reasonable request; requests should be made to Paul Leeson, Oxford Cardiovascular Clinical Research Facility, Division of Cardiovascular Medicine, Radcliffe Department of Medicine, University of Oxford, John Radcliffe Hospital, Oxford, UK (paul.leeson@cardiov.ox.ac.uk).

## Lead author biography

Paul Leeson is Professor of Cardiovascular Medicine and Fellow of Wolfson College at Oxford University. He is a Consultant Cardiologist and leads the Oxford Specialist Hypertension Clinic. His research group is interested in understanding and managing cardiovascular risk in younger people, in particular longer-term risks related to pregnancy complications. They have pioneered the application of computational modelling and artificial intelligence to clinical cardiology imaging research and work on some of the largest imaging studies in the world. Innovations arising from the work now underpin regulatory cleared diagnostic aids that are in use in hospitals in the USA and Europe.

## References

[1] Lewington S, Clarke R, Qizilbash N, Peto R, Collins R; Prospective Studies Collaboration. Age-specific relevance of usual blood pressure to vascular mortality: a meta-analysis of individual data for one million adults in 61 prospective studies. Lancet 2002;360:1903–13. 12493255 10.1016/s0140-6736(02)11911-8

[2] Bergman EM, Henriksson KM, Åsberg S, Farahmand B, Terént A. National registry-based case-control study: comorbidity and stroke in young adults. Acta Neurol Scand 2015;131:394–9. 25684429 10.1111/ane.12265

[3] Leeson P. Hypertension and cardiovascular risk in young adult life: insights from CAVI. Eur Heart J Suppl 2017;19:B24–9.

[4] Allen NB, Siddique J, Wilkins JT, Shay C, Lewis CE, Goff DC et al Blood pressure trajectories in early adulthood and subclinical atherosclerosis in middle age. JAMA 2014;311:490–7. 24496536 10.1001/jama.2013.285122 PMC4122296

[5] Hinton TC, Adams ZH, Baker RP, Hope KA, Paton JFR, Hart EC et al Investigation and treatment of high blood pressure in young people. Hypertension 2020;75:16–22. 31735086 10.1161/HYPERTENSIONAHA.119.13820

[6] Deedwania PC. The progression from hypertension to heart failure. Am J Hypertens 1997;10:280S–8S. 9366285 10.1016/s0895-7061(97)00335-x

[7] Drazner MH. The progression of hypertensive heart disease. Circulation 2011;123:327–34. 21263005 10.1161/CIRCULATIONAHA.108.845792

[8] Williams B, Mancia G, Spiering W, Agabiti Rosei E, Azizi M, Burnier M et al ESC/ESH guidelines for the management of arterial hypertension. Eur Heart J 2018;39:3021–104. 30165516 10.1093/eurheartj/ehy339

[9] Whelton PK, Carey RM, Aronow WS, Casey DE, Collins KJ, Dennison Himmelfarb C et al 2017 ACC/AHA/AAPA/ABC/ACPM/AGS/APhA/ASH/ASPC/NMA/PCNA guideline for the prevention, detection, evaluation, and management of high blood pressure in adults: executive summary: a report of the American College of Cardiology/American Heart Association Task Force on Clinical Practice Guidelines. Circulation 2018;138:e426–83. 30354655 10.1161/CIR.0000000000000597

[10] Cameli M, Lisi M, Focardi M, Reccia R, Natali BM, Sparla S et al Left atrial deformation analysis by speckle tracking echocardiography for prediction of cardiovascular outcomes. Am J Cardiol 2012;110:264–9. 22497676 10.1016/j.amjcard.2012.03.022

[11] Santos AB, Roca GQ, Claggett B, Sweitzer NK, Shah SJ, Anand IS et al Prognostic relevance of left atrial dysfunction in heart failure with preserved ejection fraction. Circ Heart Fail 2016;9:e002763. 27056882 10.1161/CIRCHEARTFAILURE.115.002763 PMC4826720

[12] Harrell FE Jr, Lee KL, Mark DB. Multivariable prognostic models: issues in developing models, evaluating assumptions and adequacy, and measuring and reducing errors. Stat Med 1996;15:361–87. 8668867 10.1002/(SICI)1097-0258(19960229)15:4<361::AID-SIM168>3.0.CO;2-4

[13] Gupta A, Bar-Joseph Z. Extracting dynamics from static cancer expression data. IEEE/ACM Trans Comput Biol Bioinform 2008;5:172–82. 18451427 10.1109/TCBB.2007.70233

[14] Campbell KR, Yau C. Uncovering pseudotemporal trajectories with covariates from single cell and bulk expression data. Nat Commun 2018;9:2442. 29934517 10.1038/s41467-018-04696-6 PMC6015076

[15] Magwene PM, Lizardi P, Kim J. Reconstructing the temporal ordering of biological samples using microarray data. Bioinformatics 2003;19:842–50. 12724294 10.1093/bioinformatics/btg081

[16] Iturria-Medina Y, Khan AF, Adewale Q, Shirazi AH. Blood and brain gene expression trajectories mirror neuropathology and clinical deterioration in neurodegeneration. Brain 2020;143:661–73. 31989163 10.1093/brain/awz400 PMC7009530

[17] Iturria-Medina Y, Carbonell F, Assadi A, Adewale Q, Khan AF, Baumeister TR et al Integrating molecular, histopathological, neuroimaging and clinical neuroscience data with NeuroPM-box. Commun Biol 2021;4:614. 34021244 10.1038/s42003-021-02133-x PMC8140107

[18] Williamson W, Lewandowski AJ, Forkert ND, Griffanti L, Okell TW, Betts J et al Association of cardiovascular risk factors with MRI indices of cerebrovascular structure and function and white matter hyperintensities in young adults. JAMA 2018;320:665–73. 30140877 10.1001/jama.2018.11498 PMC6142949

[19] Williamson W, Lewandowski AJ, Huckstep OJ, Lapidaire W, Ooms A, Tan C et al Effect of moderate to high intensity aerobic exercise on blood pressure in young adults: the TEPHRA open, two-arm, parallel superiority randomized clinical trial. EClinicalMedicine 2022;48:101445. 35706495 10.1016/j.eclinm.2022.101445 PMC9112102

[20] Mohamed A, Marciniak M, Williamson W, Huckstep OJ, Lapidaire W, McCance A et al Association of systolic blood pressure elevation with disproportionate left ventricular remodeling in very preterm-born young adults: the preterm heart and elevated blood pressure. JAMA Cardiol 2021;6:821. 33978675 10.1001/jamacardio.2021.0961 PMC8117059

[21] Lewandowski AJ, Levy PT. Exploring the cardiac phenotypes of prematurity. JAMA Cardiol 2021;6:361. 10.1001/jamacardio.2020.6056 33263728

[22] Mohamed A, Lamata P, Williamson W, Alsharqi M, Tan CMJ, Burchert H et al Multimodality imaging demonstrates reduced right-ventricular function independent of pulmonary physiology in moderately preterm-born adults. JACC Cardiovasc Imaging 2020;13:2046–8. 32417327 10.1016/j.jcmg.2020.03.016 PMC7477490

[23] Harkness A, Ring L, Augustine DX, Oxborough D, Robinson S, Sharma V. Normal reference intervals for cardiac dimensions and function for use in echocardiographic practice: a guideline from the British Society of Echocardiography. Echo Res Pract 2020;7:G1–G18. 32105051 10.1530/ERP-19-0050 PMC7040881

[24] Lang RM, Badano LP, Mor-Avi V, Afilalo J, Armstrong A, Ernande L et al Recommendations for cardiac chamber quantification by echocardiography in adults: an update from the American Society of Echocardiography and the European Association of Cardiovascular Imaging. Eur Heart J Cardiovasc Imaging 2015;16:233–71. 25712077 10.1093/ehjci/jev014

[25] Badano LP, Kolias TJ, Muraru D, Abraham TP, Aurigemma G, Edvardsen T et al Standardization of left atrial, right ventricular, and right atrial deformation imaging using two-dimensional speckle tracking echocardiography: a consensus document of the EACVI/ASE/Industry Task Force to standardize deformation imaging. Eur Heart J Cardiovasc Imaging 2018;19:591–600. 29596561 10.1093/ehjci/jey042

[26] Street JO, Carroll RJ, Ruppert D. A note on computing robust regression estimates via iteratively reweighted least squares. Am Stat 1988;42:152–4.

[27] Welch JD, Hartemink AJ, Prins JF. SLICER: inferring branched, nonlinear cellular trajectories from single cell RNA-seq data. Genome Biol 2016;17:106. 27215581 10.1186/s13059-016-0975-3 PMC4877799

[28] Abid A, Zhang MJ, Bagaria VK, Zou J. Exploring patterns enriched in a dataset with contrastive principal component analysis. Nat Commun 2018;9:2134. 29849030 10.1038/s41467-018-04608-8 PMC5976774

[29] Abdi H, Williams LJ. Principal component analysis. WIREs Comput Stat 2010;2:433–59.

[30] Mayet J, Hughes A. Cardiac and vascular pathophysiology in hypertension. Heart 2003;89:1104–9. 12923045 10.1136/heart.89.9.1104 PMC1767863

[31] Katz DH, Deo RC, Aguilar FG, Selvaraj S, Martinez EE, Beussink-Nelson L et al Phenomapping for the identification of hypertensive patients with the myocardial substrate for heart failure with preserved ejection fraction. J Cardiovasc Trans Res 2017;10:275–84. 10.1007/s12265-017-9739-z 28258421

[32] Shah SJ, Katz DH, Selvaraj S, Burke MA, Yancy CW, Gheorghiade M et al Phenomapping for novel classification of heart failure with preserved ejection fraction. Circulation 2015;131:269–79. 25398313 10.1161/CIRCULATIONAHA.114.010637 PMC4302027

[33] Nwabuo CC, Vasan RS. Pathophysiology of hypertensive heart disease: beyond left ventricular hypertrophy. Curr Hypertens Rep 2020;22:11. 32016791 10.1007/s11906-020-1017-9

[34] Todaro MC, Choudhuri I, Belohlavek M, Jahangir A, Carerj S, Oreto L et al New echocardiographic techniques for evaluation of left atrial mechanics. Eur Heart J Cardiovasc Imaging 2012;13:973–84. 22909795 10.1093/ehjci/jes174 PMC3598416

[35] Modin D, Biering-Sørensen SR, Mogelvang R, Landler N, Jensen JS, Biering-Sørensen T. Prognostic value of echocardiography in hypertensive versus nonhypertensive participants from the general population. Hypertension 2018;71:742–51. 29483222 10.1161/HYPERTENSIONAHA.117.10674

[36] Kannel WB. Left ventricular hypertrophy as a risk factor in arterial hypertension. Eur Heart J 1992;13:82–8. 1396865 10.1093/eurheartj/13.suppl_d.82

[37] Bauman AE, Reis RS, Sallis JF, Wells JC, Loos RJF, Martin BW. Correlates of physical activity: why are some people physically active and others not? Lancet 2012;380:258–71. 22818938 10.1016/S0140-6736(12)60735-1

[38] Williamson W, Foster C, Reid H, Kelly P, Lewandowski AJ, Boardman H et al Will exercise advice be sufficient for treatment of young adults with prehypertension and hypertension? A systematic review and meta-analysis. Hypertension 2016;68:78–87. 27217408 10.1161/HYPERTENSIONAHA.116.07431

