# Supplementary material for: A machine learning-based score for precise echocardiographic assessment of cardiac remodelling in hypertensive young adults
Source: Eur Heart J Imaging Methods Pract. 2023 Sep 27;1(2):qyad029. doi: 10.1093/ehjimp/qyad029 (PMC10562347; doi:10.1093/ehjimp/qyad029)
Supplement: qyad029_Supplementary_Data [file qyad029_Supplementary_Data.docx]

# Supplementary materials

Alsharqi M, Lapidaire W, Lewandowski A, Leeson P, et al. **A machine learning based score for precise echocardiographic assessment of cardiac remodelling in hypertensive young adults**

# Supplementary Methods

### Study population

The study dataset comprised of cross-sectional data collected up to March 2020 in young adults with a range of blood pressures from three ethically approved studies: (1) the Young Adult Cardiovascular Health sTudy (YACHT), (2) Trial of Exercise to Prevent HypeRtension in young Adults (TEPHRA), and (3) Hypertension management in Young adults Personalised by Echocardiography and clinical Outcomes (HyperEcho). A written informed consent was obtained from all eligible participants prior their participation. The eligibility criteria for each study are listed below:

For YACHT study, participants’ eligibility was based on the following inclusion and exclusion criteria:

**Inclusion criteria:**

- Participant is willing and able to give informed consent for participation in the study.
- Male or female aged 18 to 40 years.
- Verifiable history of preterm birth or full-term birth.
- Able (in the investigator's opinion) and willing to comply with all study requirements.
- Participant is freely able to access the John Radcliffe Hospital for study visits.

**Exclusion criteria:**

- Aged < 18 years > 40 years.
- Unwilling or unable to give informed consent for participation in the study.
- Pregnant or lactating during the course of the study.
- Planning to donate blood during the study duration.
- Any significant disease or disorder which, in the opinion of the investigator, might influence the participant's ability to participate in the study.
- Contraindication to Magnetic Resonance Imaging.

For TEPHRA, participants’ eligibility was based on the following inclusion and exclusion criteria:

**Inclusion Criteria:**

- Participant is willing and able to give informed consent for participation in the study.
- Male or female aged from 18 to 35 years old.
- Verified birth history: preterm birth (< 37 weeks) or full-term birth (> 37 weeks).
- Ability to access and use computer/internet.
- Willing to complete duration of intervention, follow-up and attend study visits at the John Radcliffe Hospital.
- 24-hour awake ABP greater than 115/75 mmHg.
- Able (in the investigator's opinion) and willing to comply with all study requirements.

**Exclusion Criteria:**

- Clinic blood pressure greater than 159 mmHg systolic and/or 99 mmHg diastolic at initial screening.
- 24-hour awake ABP greater than 150 mmHg systolic and/or 95 mmHg diastolic.
- Clinic blood pressure greater than 140 mmHg systolic and/or 90 mmHg diastolic plus evidence of end organ damage secondary to hypertension.
- Pregnancy.
- Simultaneous participation in another human or clinical randomised trial (if there was any possibility of compromising health, safety, or well-being, or any possible compromise of study data).
- Unable to walk briskly on the flat for 15 minutes.
- Those maintaining levels of cardiovascular fitness and activity at or above the levels required for the intervention arm.
- Unable to attend the regular supervised exercise sessions.
- Use of beta-blockers such as atenolol or equivalent.
- Body mass index > 35 kg/m^2^.
- Major contra-indications to exercise participation.
- Evidence of cardiomyopathy.
- Evidence of inherited cardiac conduction abnormalities.
- Evidence of congenital heart disease or significant chronic disease relevant to cardiovascular status.

For HyperEcho, participants’ eligibility was based on the following inclusion and exclusion criteria:

**Inclusion Criteria:**

- Participant is willing and able to give informed consent for participation in the study.
- Male or female, aged 18 to 40 years (at the time of their appointment at the hypertension clinic).
- Referred for a Hypertension Clinic in England.

**Exclusion Criteria:**

- Unable or unwilling to give valid consent for participation in the study.

### Development of a model without left atrial strain indices

The cardiac remodelling model involves left atrial strain indices, which are not routinely performed in clinical practice. Therefore, we aimed to compare the model stability and reproducibility with a model in which the individuals did not have left atrial strain indices. The same dataset was used to develop the later model excluding left atrial indices. Model development and internal validation process were all applied using similar codes and tests used in the original model containing left atrial indices.

## Model development and internal testing


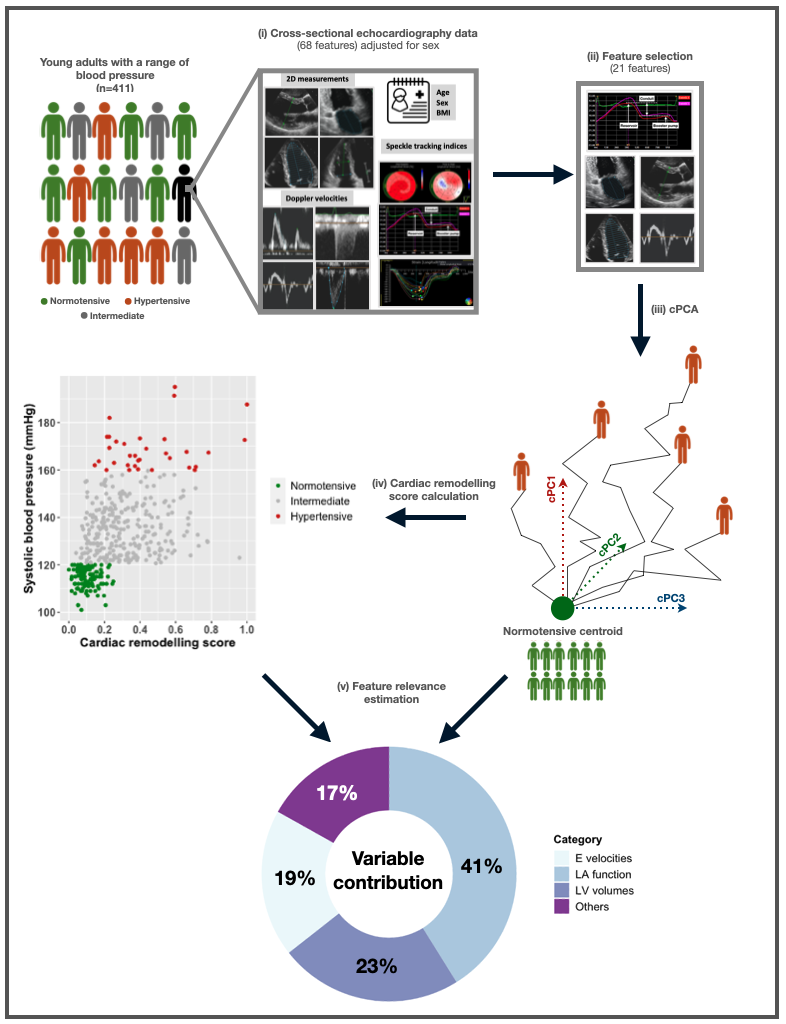


**Figure S1: The steps and outcomes of the cardiac remodelling model**

**
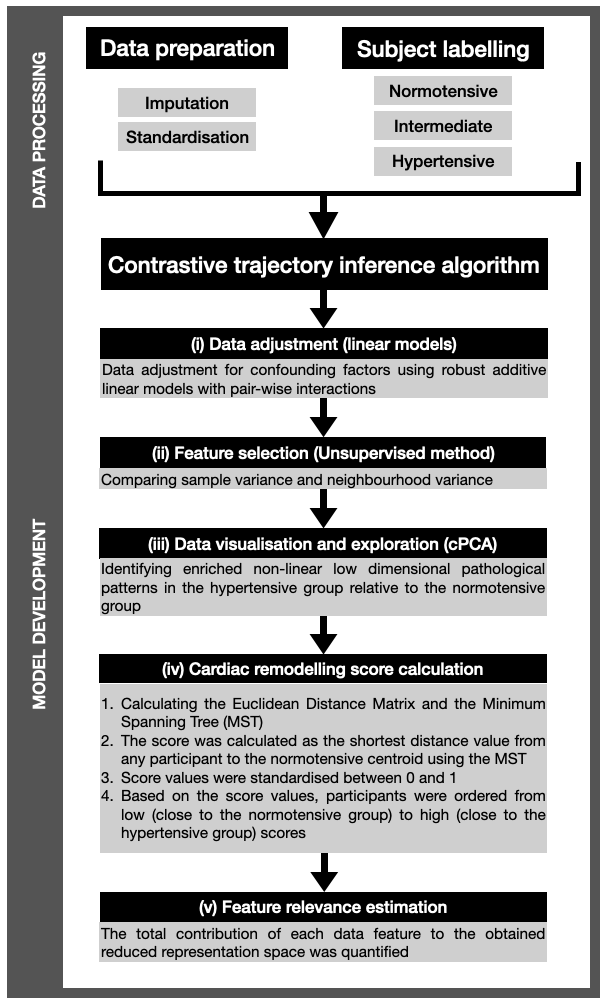
**

**Figure S2. A flow chart illustrating the steps of the cardiac remodelling model development**

# Supplementary Results

### **Figures**


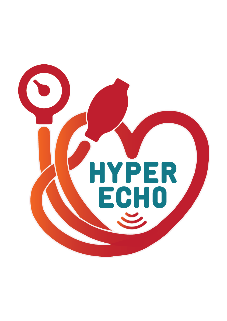

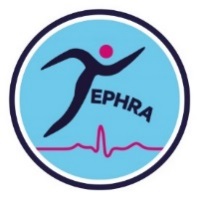

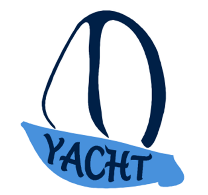


**Enrolled participants by March 2020**

**n=149**

**(63 PT and 86 FT)**

**Total recruitment**

**n=542**

**Excluded participants**

**n=117, known history of premature birth**

**n=14, >30% missing data**

**n=131**

**n=411**

**Included participants**

**n=203**

**(54 PT and 149 FT)**

**n=190**

**Figure S3: A flow diagram of the study population**

PT, preterm born adults (<37 weeks); FT, full-term born adults (>37 weeks).


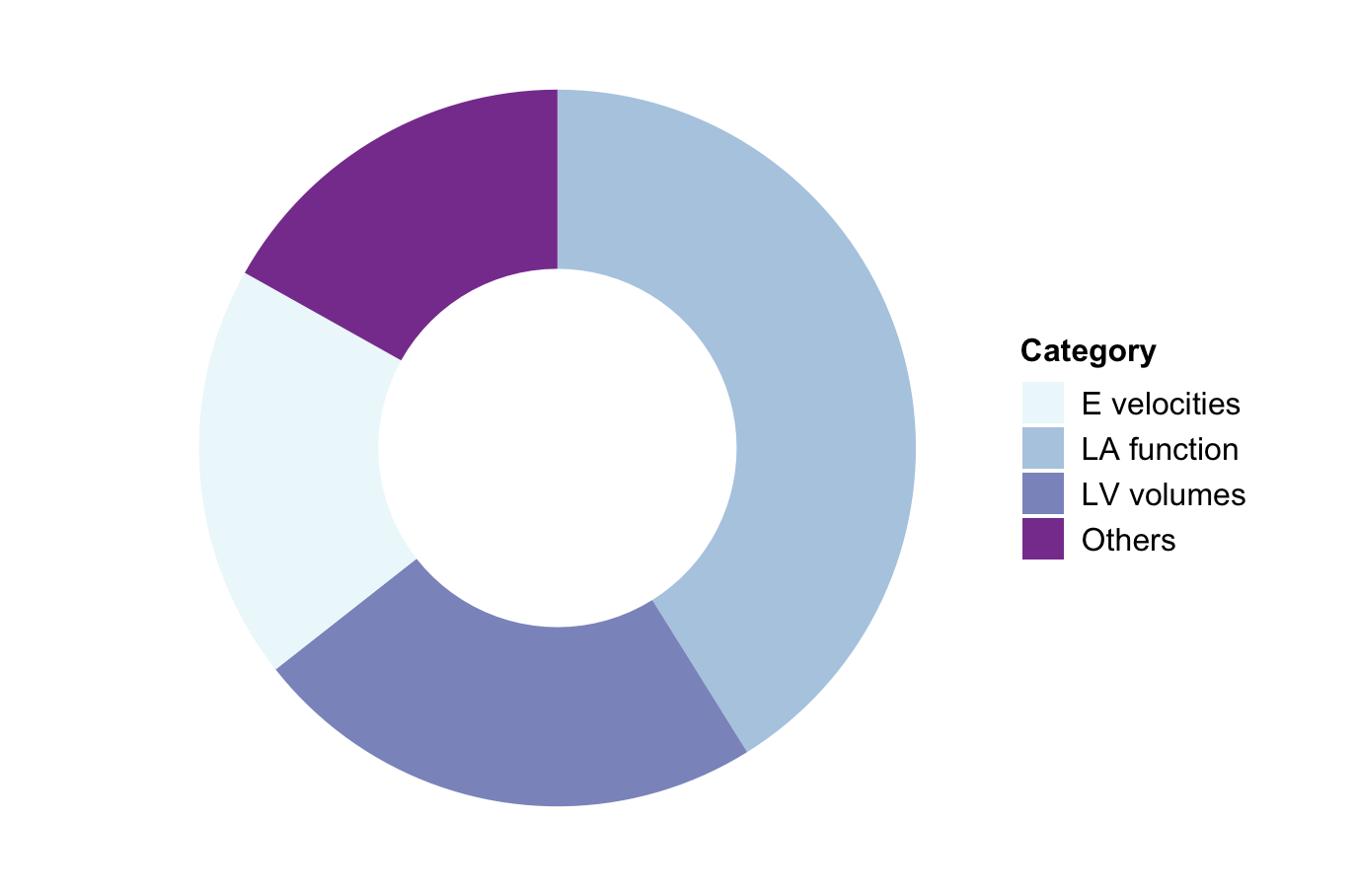


**41%**

**23%**

**19%**

**17%**

**Variable contributions**

Categories of the highest contributed variables in the model development with the percentage of contribution. About half of the model development was from the left atrial function (41%), followed by left ventricular volumes (23%) and the E velocities (19%). The remaining variables contribution was calculated at 17% of the total model development.

LA, left atrium; LV, left ventricle; EDV, end diastolic volume; ESV, end systolic volume; SV, stroke volume; bp, biplane; 4ch, four-chamber view; 2ch, two-chamber view.

| **LA 41%** | **LV 23%** | **E velocities 19%** | **Others 17%** |
| --- | --- | --- | --- |
| Conduit bp – 6.6% | Systolic diameter – 9.2% | E/e’_Average_ – 6.3% | The remaining 47 variables |
| Conduit 4ch – 6.03% | EDV 2ch – 3.5% | E/e’_medial_ – 4.5% |  |
| Reservoir bp – 5.5% | EDV bp – 3.4% | E/e’_lateral_ – 3.8% |  |
| Conduit 2ch – 4.8% | SV 2ch – 2.9% | e’_medial_ – 2.5% |  |
| Reservoir 2ch – 4.1% | ESV bp – 2.5% | e’_lateral_ – 1.6% |  |
| Pump bp – 4.04% | ESV 4ch – 1.7% |  |  |
| Pump 2ch – 3.1% |  |  |  |
| Reservoir 4ch – 3.1% |  |  |  |
| Volume bp – 2.2% |  |  |  |
| Pump 4ch – 1.6% |  |  |  |

**Figure S4. Variable contributions to the computational cardiac remodelling model**


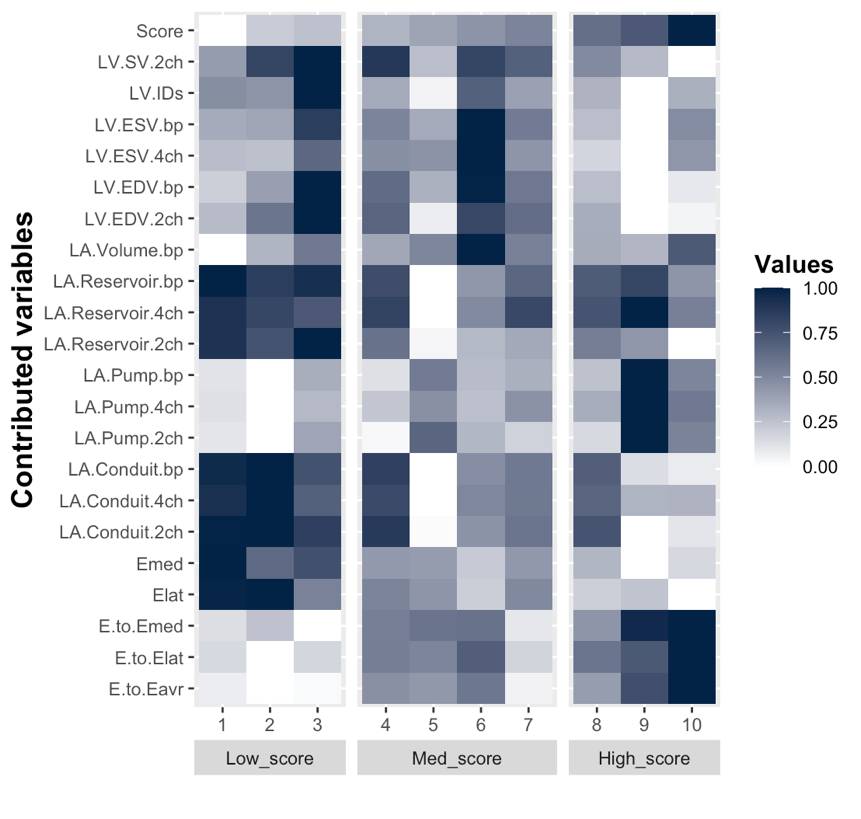


**Figure S5: The pattern of changes of individual variables throughout the spectrum of the cardiac remodelling score.**

This figure illustrates the pattern of remodelling in individual variables from health to disease. The mean value for each variable throughout the spectrum of the cardiac remodelling score was demonstrated in this heatmap. Left atrial reservoir and conduit function appear to have the same pattern as the e’ medial and lateral velocities, in which they decrease as the score progresses. In contrast, E/e’ ratios and the left atrial pump function have a similar pattern of remodelling.

### **Tables**

| **Table S1: Baseline clinical characteristics for each group of the study cohort** | | | |
| --- | --- | --- | --- |
|  | **Normotensive**  **n=111** | **Intermediate**  **n=267** | **Hypertensive**  **n=33** |
| **Age (years)** | 25.9 ± 4.2 (20) | 29.5 ± 5.7 (22) | 34.5 ± 5 (22) |
| **Male, n (%)** | 51 (46) | 133 (49.8) | 12 (36.4) |
| **Height (cm)** | 171.6 ± 9.3 (47) | 173.5 ± 10.3 (57) | 174.1 ± 10 (40) |
| **Weight (kg)** | 69.2 ± 11.5 (58) | 80.9 ± 17.8 (116) | 100.7 ± 23.4 (99) |
| **Body mass index (kg/m^2^)** | 23.6 ± 3.1 (15) | 26.9 ± 4.9 (31.7) | 31.9 ± 5.7 (22.1) |
| **Body surface area (m^2^)** | 1.8 ± 0.2 (1) | 1.9 ± 0.2 (1) | 2.2 ± 0.1 (0.5) |
| **Systolic blood pressure (mmHg)** | 114.4 ± 4.3 (19) | 135.2 ± 10 (39) | 168.7 ± 9.1 (35) |
| **Diastolic blood pressure (mmHg)** | 69.6 ± 6.6 (31) | 84 ± 10.1 (52) | 102.9 ± 9.5 (34.7) |
| **Cholesterol level (mmol/L)** | 4.2 ± 0.9 (6) | 4.6 ± 1.2 (9.4) | 5.1 ± 1 (4.5) |
| **HDL level (mmol/L)** | 1.4 ± 0.3 (1.2) | 1.3 ± 0.3 (2.6) | 1.2 ± 0.4 (1.6) |
| **LDL level (mmol/L)** | 2.5 ± 0.6 (2.5) | 2.8 ± 0.8 (5) | 3.2 ± 0.8 (3.3) |
| **Triglycerides level (mmol/L)** | 1 ± 0.7 (5) | 1.3 ± 1 (5.02) | 1.5 ± 0.8 (2.2) |
| **Cholesterol to HDL ratio** | 3.1 ± 0.8 (5.7) | 3.6 ± 1.3 (10.7) | 4.3 ± 1.1 (4) |
| **Blood Glucose level (mmol)** | 5.05 ± 2.7 (25.5) | 6.3 ± 5.7 (28.8) | 10.6 ± 10.3 (27.8) |
| **Smokers, n (%)** | 9 (8.1) | 44 (16.5) | 5 (15.2) |
| **On antihypertension medication, n (%)** | 0 (0) | 94 (35.2) | 25 (75.7) |
| Numeric data is presented as mean ± standard deviation and (range), and categorical data is presented as number of participants and (percentage). | | | |

| **Table S2: Echocardiography characteristics for each group of the study cohort** | | | |
| --- | --- | --- | --- |
|  | **Normotensive**  **n=111** | **Intermediate**  **n=267** | **Hypertensive**  **n=33** |
| **Heart rate (bpm)** | 60.3 ± 10.7 | 66.1 ± 11.7 | 66.8 ± 9.5 |
| ***Left ventricular structure and function*** | | | |
| **Diastolic diameter (cm)** | 4.6 ± 0.5 | 4.7 ± 0.5 | 4.8 ± 0.5 |
| **Systolic diameter (cm)** | 3.1 ± 0.4 | 3.1 ± 0.4 | 3.1 ± 0.6 |
| **Interventricular septum thickness (cm)** | 0.8 ± 0.2 | 0.9 ± 0.2 | 1.1 ± 0.2 |
| **Inferolateral (posterior) wall thickness (cm)** | 0.9 ± 0.2 | 0.9 ± 0.2 | 1.1 ± 0.2 |
| **Relative wall thickness** | 0.37 ± 0.07 | 0.4 ± 0.09 | 0.47 ± 0.1 |
| **Mass index (g/m^2^)** | 63.7 ± 14.5 | 74 ± 17.2 | 88.8 ± 22.5 |
| **Biplane end diastolic volume (ml)** | 93.9 ± 24.3 | 100.1 ± 25.3 | 113.2 ± 28.7 |
| **Biplane end systolic volume (ml)** | 35.5 ± 11.4 | 36.9 ± 11.2 | 43.4 ± 15.6 |
| **Biplane ejection fraction (%)** | 62.8 ± 5.2 | 63.3 ± 5.7 | 62.4 ± 5.5 |
| **Biplane stroke volume (ml)** | 58.2 ± 14.7 | 63.3 ± 16.6 | 69.9 ± 15.1 |
| **Mitral valve E velocity (cm/sec)** | 81.3 ± 17.1 | 78.1 ± 14.7 | 75.7 ± 18.9 |
| **Mitral valve A velocity (cm/sec)** | 48.5 ± 12.1 | 54.6 ± 12.2 | 59.8 ± 12.1 |
| **E/A ratio** | 1.7 ± 0.5 | 1.5 ± 0.4 | 1.3 ± 0.5 |
| **Mitral valve deceleration time (sec)** | 0.2 ± 0.04 | 0.2 ± 0.04 | 0.2 ± 0.03 |
| **Lateral e’ velocity (cm/sec)** | 16.8 ± 3.4 | 15.3 ± 3.9 | 11.8 ± 2.8 |
| **Septal e’ velocity (cm/sec)** | 11.9 ± 2.2 | 10.4 ± 2.2 | 8.4 ± 2.4 |
| **E/e’_Lateral_ (cm/sec)** | 5 ± 1.4 | 5.4 ± 1.6 | 7 ± 2.7 |
| **E/e’_Septal_ (cm/sec)** | 7 ± 1.6 | 7.7 ± 1.8 | 9.5 ± 3.3 |
| **Global longitudinal strain (%)** | -21.2 ± 2.5 | -20.2 ± 2.1 | -18.6 ± 1.8 |
| ***Left atrial structure and function*** | | | |
| **Biplane left atrial volume (ml)** | 36.7 ± 10 | 41.3 ± 11.6 | 47.5 ± 14.6 |
| **Reservoir function – Peak longitudinal strain (%)** | 39.3 ± 8 | 36.2 ± 7.4 | 32.4 ± 5.8 |
| **Booster pump function – Peak contraction strain (%)** | 9.3 ± 4.9 | 9.6 ± 5.4 | 11.7 ± 5.4 |
| **Conduit function – The difference (%)** | 30 ± 7.3 | 26.5 ± 7.4 | 20.6 ± 5.2 |
| ***Right heart structure and function*** | | | |
| **RV Basal diameter (cm)** | 3.6 ± 0.5 | 3.5 ± 0.5 | 3.5 ± 0.4 |
| **RV Mid diameter (cm)** | 2.6 ± 0.5 | 2.6 ± 0.5 | 2.4 ± 0.4 |
| **RV Length (cm)** | 7 ± 1 | 7 ± 0.8 | 7 ± 0.7 |
| **TAPSE (cm)** | 2.1 ± 0.3 | 2.2 ± 0.3 | 2.2 ± 0.4 |
| **RV s’ velocity (cm/sec)** | 12.3 ± 1.7 | 12.2 ± 2 | 13 ± 1.9 |
| **RA volume (ml)** | 37.4 ± 12.7 | 37.1 ± 12.7 | 37.6 ± 13.1 |
| Data is presented as mean ± standard deviation. Bpm, beat per minute; RV, right ventricle; RA, right atrium; TAPSE, tricuspid annular plane systolic excursion. | | | |

| **Table S3. Clinical and echocardiography characteristics before and after a 16-week exercise intervention** | | | |
| --- | --- | --- | --- |
|  | **Exercise intervention participants**  **(n=60)** | |  |
|  | **Pre-intervention** | **Post-intervention** | ***p* value** |
| **Cardiac remodelling score** | 0.24 ± 0.14 | 0.23 ± 0.13 | 0.278 |
| **Body mass index (kg/m^2^)** | 24.93 ± 3.64 | 24.66 ± 3.69 | 0.047 |
| ***Left ventricular structure and function*** | | | |
| **Diastolic diameter (cm)** | 4.64 ± 0.46 | 4.71 ± 0.38 | 0.114 |
| **Systolic diameter (cm)** | 3.01 ± 0.36 | 3.05 ± 0.34 | 0.36 |
| **Interventricular septum thickness (cm)** | 0.8 ± 0.14 | 0.8 ± 0.16 | 0.866 |
| **Inferolateral wall thickness (cm)** | 0.88 ± 0.15 | 0.87 ± 0.17 | 0.775 |
| **Relative wall thickness** | 0.38 ± 0.08 | 0.37 ± 0.07 | 0.295 |
| **Mass index (g/m^2^)** | 69.1 ± 15.02 | 70.56 ± 16.07 | 0.376 |
| **Biplane end diastolic volume (ml)** | 95.1 ± 25.78 | 92.42 ± 19.29 | 0.276 |
| **Biplane end systolic volume (ml)** | 34.72 ± 10.02 | 33.47 ± 9 | 0.176 |
| **Biplane ejection fraction (%)** | 63.38 ± 4.89 | 63.85 ± 5.07 | 0.485 |
| **Biplane stroke volume (ml)** | 60.5 ± 17.61 | 58.72 ± 13 | 0.35 |
| **Mitral valve E velocity (cm/sec)** | 79.28 ± 16.19 | 79.33 ± 15.73 | 0.981 |
| **Mitral valve A velocity (cm/sec)** | 51.82 ± 10.97 | 49.11 ± 11.57 | 0.095 |
| **E/A ratio** | 1.58 ± 0.4 | 1.65 ± 0.47 | 0.22 |
| **Mitral valve deceleration time (sec)** | 0.18 ± 0.03 | 0.19 ± 0.03 | 0.142 |
| **Lateral e’ velocity (cm/sec)** | 16.86 ± 3.49 | 16.42 ± 2.82 | 0.31 |
| **Septal e’ velocity (cm/sec)** | 11.23 ± 2.1 | 10.91 ± 2.13 | 0.178 |
| **E/e’_Lateral_ (cm/sec)** | 4.84 ± 1.12 | 4.9 ± 1.08 | 0.721 |
| **E/e’_Septal_ (cm/sec)** | 7.26 ± 1.49 | 7.41 ± 1.5 | 0.47 |
| **Global longitudinal strain (%)** | -21.81 ± 1.71 | -22.09 ± 2.15 | 0.324 |
| ***Left atrial structure and function*** | | | |
| **Biplane left atrial volume (ml)** | 38.32 ± 10.31 | 38.50 ± 9.73 | 0.856 |
| **Reservoir function (%)** | 37.04 ± 5.87 | 36.18 ± 6.04 | 0.277 |
| **Booster pump function (%)** | 8.93 ± 4.97 | 9.03 ± 4.6 | 0.883 |
| **Conduit function (%)** | 28.1 ± 6.11 | 27.14 ± 5.16 | 0.246 |
| ***Right heart structure and function*** | | | |
| **RV Basal diameter (cm)** | 3.66 ± 0.51 | 3.6 ± 0.5 | 0.307 |
| **RV Mid diameter (cm)** | 2.72 ± 0.51 | 2.59 ± 0.52 | 0.071 |
| **RV Length (cm)** | 6.82 ± 0.99 | 6.84 ± 0.85 | 0.897 |
| **TAPSE (cm)** | 2.1 ± 0.26 | 2.13 ± 0.31 | 0.475 |
| **RV s’ velocity (cm/sec)** | 12.24 ± 1.61 | 12.17 ± 1.68 | 0.723 |
| **RA volume (ml)** | 36.99 ± 10.67 | 38.38 ± 13.99 | 0.292 |
| Data is presented as mean ± standard deviation. RV, right ventricle; RA, right atrium; TAPSE, tricuspid annular plane systolic excursion. | | | |
